# Adding capacity and compassion to Canada’s mental health crisis: The impact of kids help phone’s crisis responder volunteers

**DOI:** 10.1371/journal.pmen.0000321

**Published:** 2025-06-05

**Authors:** Alisa Simon, Andréanne Deschamps, Kaylea Walsh, Tamar Brannigan

**Affiliations:** Kids Help Phone, University Ave, Toronto, Ontario, Canada; PLOS: Public Library of Science, UNITED KINGDOM OF GREAT BRITAIN AND NORTHERN IRELAND

## Introduction

Canada is experiencing a severe youth mental health crisis, with a 12% decline in mental health among youth aged 12–17, and only 35% of those in need being able to access professional help [1].

While Kids Help Phone (KHP)’s Crisis Responders are not traditionally considered public safety personnel (PSP), they share many similar challenges: operating in high-stakes situations, managing emotional intensity, and providing essential support to vulnerable populations.

For 35 years, KHP has provided critical support to young people, evolving from a phone helpline to an e-mental health ecosystem with a 147% increase in service growth since 2020, reaching youth more that 21 million times. A crucial aspect of this transformation has been training and deploying over 10,000 volunteer texting Crisis Responders (CRs) since 2018, forming what is likely to be Canada’s largest mental health training program. The contributions of these volunteers extend beyond immediate support, to building mental health capacity across communities in Canada.

This Opinion is informed by the findings from a 2024 survey of 885 KHP Crisis Responders, highlighting how these volunteers have met the pressing needs of youth in crisis, which could potentially inform similar initiatives in other communities. We will shine a spotlight on the themes of skill development through volunteer training and the ripple effects of these skills in strengthening both the volunteers and their communities.

### Skills development and community impact

Crisis Responder volunteers must pass a rigorous training, which equips them with necessary skills in crisis de-escalation, active listening, and empathetic communication over text. This training model not only equips volunteers but serves as an innovative framework that could inform broader mental health support systems, including strategies for PSP working in mental health. As a result of the training and experience as a KHP volunteer, 80% of CRs report significant growth in their ability to identify and respond to crises, with 86% noting substantial improvement in active listening.

Perhaps most importantly, the skills they learn are not confined to the texting platform where they volunteer. Nearly 78% of volunteers feel confident supporting people in distress in their everyday lives, extending their impact far beyond the texting service. In fact, survey data revealed that 87% of volunteers have identified someone in need of mental health support outside their role, often stepping in to provide direct assistance. This creates a ripple effect, impacting the lives of family and community members across Canada. This cascade of support builds a network of informal mental health advocates, helping to normalize mental health conversations and break down stigmas in local communities from coast to coast to coast.

### Personal growth and purpose

The benefits of being a KHP Crisis Responder extends beyond skill-building. Many CRs reported that the experience is personally transformative with 77% finding greater purpose in their lives through their CR role. In addition, eight in ten volunteers report a strong sense of accomplishment from their volunteer experience.

This sense of purpose is often rooted in the lived experience of volunteers. In fact, nearly 40% of CRs reported personal connection to mental health, either through their own journeys or those of loved ones. This suggests that many volunteers are driven by a deep understanding of mental health challenges and a desire to provide the support they may have once needed themselves.

“*When I was in my senior year of high school I was depressed and very lonely,”* one volunteer told us*. “But I was very fortunate to receive great help and support from my family and friends. I would also like for others to receive support when they need it the most, because I know how hard and dark things can feel.*” Like many first responders, volunteers often draw from their own lived experiences to support others, finding meaning and healing in the process [2]. This dual impact—helping others while fostering personal growth—echoes the psychological resilience-building seen in other caregiving professions.

While supporting young people through the texting service, providing crisis support also helps CRs directly. Sixty-four percent report improved self-care practices and enhanced positive feelings about themselves as a direct result of their volunteer experience.

### Inspiring future leaders

KHP’s CR training is also helping to shape the next generation of mental health professionals and support. Nearly 80% of volunteers state that the experience inspired them to pursue careers in helping professions, with 47% reporting shifts in their career aspirations toward more purpose-driven work. This demonstrates how e-mental health platforms like KHP can serve as a talent pipeline, strengthening Canada’s mental health workforce while fostering a culture of community care.

For some, volunteering has translated into tangible outcomes. Over one-third of CRs credit the program for helping them secure jobs or access further training, ultimately contributing to long-term capacity-building in Canada’s mental health workforce.

### Building a more mentally healthy canada

The impact KHP’s Crisis Responders extends beyond the individual texting conversations they take. By training thousands of volunteers in crisis intervention, the program is creating a network of skilled mental health advocates embedded in communities across the country.

These volunteers provide immediate support to hundreds of thousands of people each year while also contributing to changing the broader mental health landscape. In fact, over 50% of CRs stated they feel empowered to advocate for mental health policies, creating awareness and influencing change in schools, workplaces, and healthcare settings. These efforts position Crisis Responders not only as service providers but also as catalysts for broader societal change, influencing mental health policies and improving community support systems.

## Conclusion

As Canada faces an escalating youth mental health crisis, programs like Kids Help Phone’s Crisis Responder initiative offer a beacon of hope. Leveraging innovative text-based technology, this program enables scaled support through simultaneous conversations and enhanced clinical oversight. KHP’s unique training and model empower volunteers to provide immediate, meaningful assistance to youth in need. By investing in initiatives like these, Canada can expand this scalable approach, applying its success to other critical areas of mental health care and public safety.

For volunteers, the experience is life-changing, offering skills, purpose, and opportunities for personal and professional growth. For society, the program represents a scalable, sustainable model for addressing the mental health crisis—one conversation at a time.

As Crisis Responders carry their skills and compassion into their communities, workplaces, and beyond, they become catalysts for change. They prove that the power of connection can transform lives and build a stronger, more resilient Canada.

Every day, Kids Help Phone’s Crisis Responders answer the texts that transform connection into courage, compassion into change, and build a Canada where all youth are empowered to feel out loud.
